# NOVEL BIOMARKERS OF BIOLOGICAL AGE IN THE HEALTH AND RETIREMENT STUDY

**DOI:** 10.1093/geroni/igz038.1610

**Published:** 2019-11-08

**Authors:** Bharat Thyagarajan, Morgan E Levine

**Affiliations:** 1 Department of Laboratory Medicine and Pathology University of Minnesota; Minneapolis, Minnesota, United States; 2 Yale University School of Medicine, New Haven, Connecticut, United States

## Abstract

This symposium presents early results on epigenetic, transcriptomic and other aging biomarkers such as telomere length and mitochondrial DNA copy number from the Health and Retirement Study that allows a detailed examination of the biological pathways through which socioeconomic conditions influence the human aging process. In 2016 HRS collected 6 tubes of blood from people who completed the 2016 interview, had been in the sample at the prior wave and were not in a nursing home (n=9,973) to maintain a nationally-representative sample. These blood samples were analyzed for novel biomarkers of aging that included global methylation arrays, whole transcriptome sequencing, telomere length and mitochondrial DNA copy number among other biomarkers that were shown to be related to both social and economic circumstances and health outcomes at older ages. This level of integration of biological data to address social disparities hasn’t been accomplished before on a large nationally-representative sample of Americans and will provide a unique opportunity to understand the biological mechanisms through which social disparities affect human health. The symposium will describe the utility of measuring novel age related biomarkers in a nationally representative population study such as HRS and the potential research opportunities that can be pursued using this publicly available resource. It will provide an overview of the measurement and distribution of epigenetic, transcriptomic and telomere length and mitochondrial DNA copy number as novel aging biomarkers. It will also describe the utility of these biomarkers in further understanding the biological underpinnings of socioeconomic differences in health and mortality.

